# The Efficacy and Safety of Using Chamomile Products During Pregnancy and the Postpartum Period

**DOI:** 10.7759/cureus.81527

**Published:** 2025-03-31

**Authors:** Tess Ferguson, Barbara Gordon

**Affiliations:** 1 Obstetrics and Gynecology, Idaho College of Osteopathic Medicine, Meridian, USA; 2 Nutrition and Dietetics, Idaho State University, Meridian, USA

**Keywords:** and pregnancy, chamomile, complementary and alternative medicine (cam), herbal remedies, homeopathic treatments, labor pain managment, nausea and vomiting in pregnancy, postpartum depression, postpartum mental health, womens health

## Abstract

Herbal remedies have been a mainstay of medicine for thousands of years. This systematic literature review investigated the efficacy and safety of chamomile herbal products among peripartum or postpartum women. Four peer-reviewed databases were searched through June 2024. The Preferred Reporting Items for Systematic Reviews and Meta-Analyses (PRISMA) protocol was implemented, and the quality of studies was assessed using Cochrane Risk-of-Bias Assessment tools.

A total of 23 studies (16 clinical trials and seven observational studies involving 2,065 women from nine countries) were included in this review. The development of clinical practice recommendations on using chamomile products during pregnancy is not feasible based on the available evidence, indicating the need for randomized, double-blind placebo control studies with larger study populations and consistent study protocols (e.g., type and dosage of chamomile ingested). Limitations of this review include its small sample size and the inclusion of multiple studies by the same research teams; these findings likely reflect insights from the same cohorts of women.

Some studies reported clinically significant findings that were not statistically significant. Thus, despite weak evidence supporting the efficacy and safety of chamomile usage during pregnancy, a provider might still share the potential benefits and risks of using chamomile products with patients who use or desire to use chamomile products.

## Introduction and background

Herbal remedies have been a mainstay of medicine for the last 5000 years [1], and people still consume herbs for health reasons. Herbal product sales have increased in the last 20 years; in 2021, sales totaled more than $12 billion in the United States (US) [2]. Many studies have demonstrated widespread acceptance, particularly by women, of herbal and other natural remedies [3]; many women reported continued usage during pregnancy [4]. An Italian survey (n=150) found that 52% of women believed that complementary and alternative drugs provided safe alternatives to conventional medications during pregnancy; 62.7% thought they were just as effective [5]. Other research supports these findings; continued use of herbal products during pregnancy may be due to the perception that they are safe, less harmful than conventional medicines, and lack serious side effects [4,6,7,8]. Indeed, advertisers actively promote herbal remedies for use during pregnancy in many Western markets [7,9].

Chamomile is one of the most commonly used herbs during pregnancy [6,10,11]. Women use it to treat many ailments experienced during the peripartum and postpartum periods, including anxiety, sleeping problems, nausea, and stretch marks [6,9,12]. Chamomile has also traditionally been used to manage digestive issues, promote wound healing, encourage sedation, and relieve pain associated with arthritis and back injuries [1]. Not all patients report their use of complementary and alternative medicines (CAM) to their physicians; however, herb-drug interactions exist [9,12]. If taken in conjunction with psychotropic medications, chamomile may enhance the central nervous system effects of those pharmaceuticals [9]. Due to the presence of coumarins and the risk for additive effects, chamomile consumption is contraindicated for individuals taking anticoagulant and antiplatelet medications [13]. 

A member of the Asteraceae botanical family, chamomile is a crop grown for its dietary, cosmetic, and medicinal purposes [14]. Two species of chamomile are frequently used for medicinal purposes: German chamomile (Matricaria chamomilla) and Roman or English chamomile (Chamaemelum nobile) [14,15]. The scent of chamomile is often described as fresh, sweet, fruity, and warm [15]. Most of its therapeutic compounds are contained within the flower, which is used to make an essential oil with over 120 components, including fla­vonoids, sesquiterpenes, and coumarins [13,16]. Chamomile’s chemical composition may vary depending on the environment the plant was grown in, the content of the soil, and the genotype of the plant [16].

The main bioactive ingredient in chamomile essential oil is apigenin, which has anti-inflammatory, antioxidant, and sedative properties [16]. German chamomile has higher levels of apigenin, and it is used more often in herbal remedies [16]. Chamomile also contains α-bisabolol, which has antimicrobial properties and other components that possess analgesic, anti-cancer, anti-hypertensive, and anti-allergic characteristics [16]. Four saturated fats make up the primary metabolites of chamomile: palmitic acid, linoleic acid, oleic acid, and stearic acid [14].

Delivery modes of chamomile herbal products include capsules, teas, oils, and creams [15]. Chamomile tea is an infusion made from the plant’s flowers and chamomile capsules contain extract also made from the flowers [17,18]. Oils and creams are used in aromatherapy treatments, which include inhalation of the oil or topical applications such as massage [15]. Of note, tinctures of chamomile can contain up to 12% grain alcohol; the literature recommends avoiding herb tinctures during pregnancy [1,13].

Aim of this study

This study evaluated the existing literature on the safety and efficacy of chamomile use during the peripartum and postpartum periods of pregnancy. The strengths and limitations of the included studies are discussed. Clinically significant vs. statistically significant results emerged, and guidance on the translation of the findings into practice is offered.

This article was previously presented as a poster at the American College of Osteopathic Obstetricians and Gynecologists’ 2024 Advances in Women's Health Conference on November 2, 2024.

## Review

Methods

A systematic literature review was conducted to investigate the efficacy and safety of chamomile herbal products among peripartum or postpartum women.

Search Strategy

Four databases (CINAHL, Google Scholar, MEDLINE Ultimate, and Science Direct) were searched through June 12, 2024. For all databases except Google Scholar, Boolean search terms were employed. Google Scholar’s advanced search options were used to find keywords in article titles. Due to variations in search parameters, the search chains varied. Articles were also retrieved from bibliographies and by free-searching during July 2024. Table 1 provides a summary of search chains for each database.

**Table 1:** Search strategy employed for various databases

Database	Search chain	Results
CINAHL	((MH “Chamomile”) OR chamomile OR camomile OR mayweed OR “matricaria chamomilla”)) AND ((MH “Pregnancy”) OR (MH “Pregnancy Outcomes”) OR (MH “Pregnancy Complications”) OR (MH “Perinatal Period”) OR (MH “Postnatal Period”) OR (MH “Postnatal Care”) OR (MH “Breast Feeding”) OR Pregnancy OR Pregnancy OR gravid OR gestat* OR gestation* OR peripartum OR postpartum OR “breast feeding” OR lactating))	28
Google Scholar	Chamomile, camomile, and matricaria chamomilla combined with pregnancy, pregnant, postpartum, perinatal, breast feeding, breastfeeding, peripartum, and postnatal	23
MEDLINE Ultimate	((MH “Chamomile”) OR Chamomile OR camomile OR Mayweed OR “Matricaria chamomilla”)) AND ((MH “Pregnancy+”) OR (MH “Pregnancy Outcome+”) OR (MH “Pregnancy Complications+”) OR (MH “Perinatal care+”) OR (MH “Maternal Health+”) OR Pregnant OR Pregnancy OR Gravid OR Gestation* OR Gestat* OR Peripartum OR Postpartum OR “Breast feeding” OR lactating OR perinatal OR maternal OR (MH “Breast Feeding+”))	77
Science Direct	(chamomile OR camomile OR mayweed OR "matricaria chamomilla") AND (pregnancy OR pregnant OR peripartum OR postpartum OR "breast feeding")	460

Article Selection and Data Collection 

The author TF completed the title review and abstract review, while author BG reviewed them. Inclusion and exclusion criteria were determined via a series of discussions. Both authors completed the full text review to finalize the articles for inclusion in the study. Author, year, sample size, participant demographics, intervention, duration, and outcome measures were compiled and evaluated. Outcomes focused on the safety and efficacy findings of various modes of consumption of chamomile herbal products.

Inclusion and exclusion criteria: Inclusion criteria were peer-reviewed primary research studies (experimental and observational) published in English that provided findings of chamomile as an intervention. Secondary research, conference papers/abstracts, case reports/series, clinical guidelines, and animal/lab studies were excluded (Table 2).

**Table 2:** Inclusion criteria

Type of studies	Study subject criteria	Outcomes of interest
Peer-reviewed primary studies; experimental and observational studies	Published in the English language, providing findings of chamomile as an intervention during peripartum and postpartum periods of pregnancy	Safety and efficacy findings of various modes of consumption of chamomile herbal products

Ascertainment of Risk of Bias in Individual Studies

Quality assessments were conducted using the following Cochrane Risk-of-Bias Assessment tools: the Risk of Bias 2 (RoB 2) and Risk of Bias in Non-randomized Studies - of Exposure (ROBINS-E) [19,20]. The RoB 2 assessed the risk of bias for clinical trials across five domains: bias arising from the randomization process, deviations from intended interventions, missing outcome data, measurement of outcomes, and selection of the reported result [19]. Within each domain and overall, grading options were “low”, “some concerns”, or “high” risk of bias [19]. The ROBINS-E evaluated the risk of bias for observational studies across seven domains: bias due to confounding, bias arising from measurement of exposure, bias in selection of participants into the study/analysis, bias due to post-exposure interventions, bias due to missing data, bias arising from measurement of the outcomes, and bias in selection of the reported result [20]. Response options were “yes,” “probably yes.”, “probably no,” “no,” and “no information” [20]. TF completed the risk-of-bias assessments; BG reviewed, and disagreements were resolved via a series of discussions.

*Synthesis Process*

Themes were narratively compiled on the efficacy and safety of chamomile herbal product usage during pregnancy. Efficacy outcomes evaluated included the treatment of nausea/vomiting, pain control, sleep quality, anxiety, bowel motility, labor induction, and postpartum depression. Safety outcomes investigated were the potential effect on preterm labor/delivery, miscarriage risk, and birth weight and length. Based on Cochrane's meta-analysis guidelines, a meta-analysis was not conducted because of the high risk of bias in the observational studies and the heterogeneity of the clinical trials (product used, dosages, modes of delivery) [21].

Results

**Figure 1:**
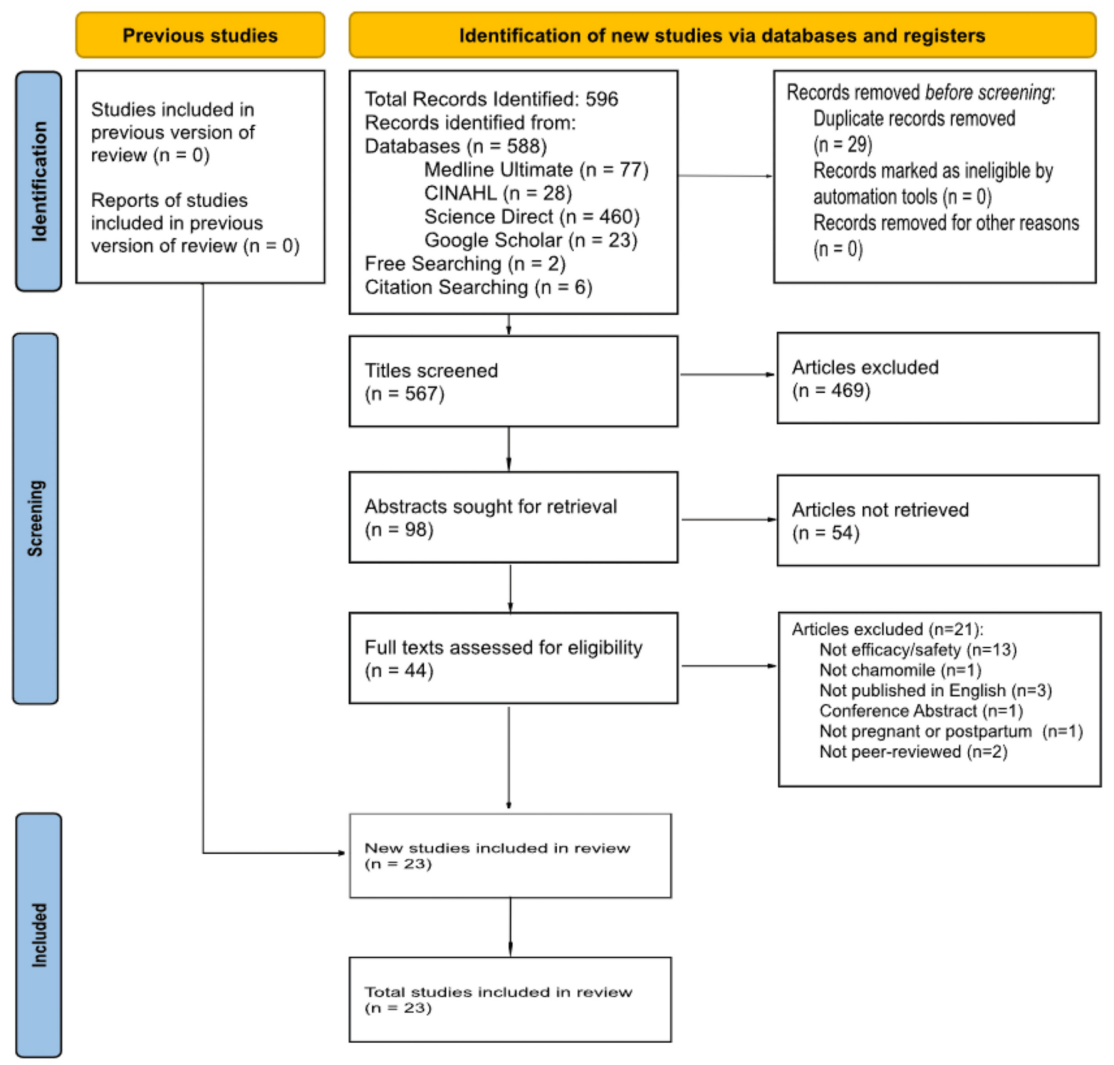
PRISMA flowchart depicting the selection of studies PRISMA: Preferred Reporting Items for Systematic Reviews and Meta-Analyses

The Preferred Reporting Items for Systematic Reviews and Meta-Analyses (PRISMA) protocol was employed for this study [22]. Figure 1 shows the PRISMA flowchart depicting the study selection process.

A total of 23 articles were included in the final analysis: 16 clinical trials and seven observational studies [6-9,12,23-40]. The included studies reflect the experiences of a combined 8,343 women (2,065 of whom used chamomile) from nine countries (Canada, Italy, Australia, Norway, Jordan, United Kingdom, Iran, Pakistan, and Taiwan) [6-9,12,23-40]. Most of the articles (19/23, 83%) provided findings on the efficacy of chamomile as an alternative therapy during various stages of pregnancy [23-40]; the other five studies reported on safety considerations [6-9,12].

Risk-of-Bias Assessment

The ROBINS-E revealed “some concern” of bias in four studies [6,7,9,12]. One study had a high risk of bias: the measured exposure (chamomile usage) was not well-characterized by the exposure metric [8]. Furthermore, Mousally et al. did not report on participant knowledge regarding the risk of the outcome, which could have affected exposure measurements [8]. The three observational studies on efficacy had a very high risk of bias [23-25]. One did not report on the exact number of women who used chamomile since the study focused on a combination of essential oils, introducing some bias for the reported result [24]. For many observational studies, a retrospective study design introduced an element of selection bias [6-9,12,23,25]. Exposure measurement was also not exact as most of the observational studies relied on participant memory of how much chamomile they used during pregnancy, and the types of products used by participants varied [6-9,12,23,25]. Despite controlling for confounding variables, uncertainty remains about how other herbal supplements and medications may have affected the results of those studies. Table 3 summarizes the Robins-E findings.

**Table 3:** Risk-of-bias analysis for observational studies on the efficacy and safety of chamomile usage during pregnancy based on ROBINS-E CH: chamomile; C/S: cesarean section; LBL: low birth length; LBW: low birth weight; M/C: miscarriage; PTB: preterm birth; PTL: preterm labor; ROBINS-E: Risk of Bias in Non-randomized Studies - of Exposure

Study	Outcome measured	Confounding variables	Measurement of exposure	Participant selection	Post-exposure interventions	Missing data	Measurement of outcome	Reported result	Overall
Safety studies									
Cuzzolin et al., 2010 [6]	M/C, PTL	Concerns	Concerns	Low	Low	Low	Concerns	Low	Concerns
Facchinetti et al., 2012 [7]	PTB, LBW	Concerns	Concerns	Concerns	Low	Low	Low	Low	Concerns
Moussally and Bérard, 2012 [8]	LBW	Concerns	High	Concerns	Low	Low	Low	Low	High
Nordeng et al., 2011 [9]	LBL, C/S	Concerns	Concerns	Concerns	Low	Low	Low	Low	Concerns
Trabace et al., 2015 [12]	PTB, LBW, LBL	Concerns	Concerns	Concerns	Low	Low	Low	Concerns	Concerns
Efficacy studies									
Khresheh, 2011 [23]	N/V	Concerns	High+	High	Low	High	High	Low	High+
Pollard, 2007 [24]	Labor pain	High	Concerns	Concerns	Low	High+	High	Concerns	High+
Forster et al., 2006 [25]	N/V, digestion, anxiety, sleep	Low	High	High+	Low	Low	Concerns	High+	High+

The RoB 2 revealed that seven RCTs had a low risk of bias [26-32], while five studies had concerns for bias [33-37], and three studies had a high risk of bias [38-40]. Many of these studies had concerns related to measurement error bias, potentially introduced due to a lack of information on the chemical composition of the chamomile [33,35-37]. The three studies with a high risk of bias were not blinded, yielding a higher risk for measurement error and reporting bias [38-40]. Table 4 provides a summary of the RoB 2 assessment.

**Table 4:** Risk-of-bias analysis for experimental studies on the efficacy and safety of chamomile usage during pregnancy employing RoB 2 BM: bowel movement; CS: cesarean section; PPD: postpartum depression; PDPH: post-dural puncture headache; RoB 2: Risk-of-bias Tool for Randomized Trials

Study	Outcome measured	Randomization process	Intervention deviation	Missing data	Measurement error	Reported result	Overall score
Efficacy studies							
Labor							
Heidari-fard et al., 2017 [28]	Anxiety	Low	Low	Low	Low	Low	Low
Heidari-fard et al., 2018 [29]	Relaxation	Low	Low	Low	Low	Low	Low
Zafar et al., 2016 [30]	Pain	Low	Low	Low	Low	Low	Low
Gholami et al., 2016 [34]	Labor induction	Low	Low	Low	Concern	Low	Concern
Eskandari et al., 2022 [38]	Anxiety, pain	Low	Low	Low	High	Concern	High
Eskandari et al., 2023 [39]	Anxiety, pain	Low	Low	Low	High	Concern	High
Postoperative							
Aradmehr et al., 2017 [27]	Episiotomy pain	Low	Low	Low	Low	Low	Low
Najafi et al., 2017 [31]	CS pain	Low	Low	Low	Low	Low	Low
Zardosht et al., 2021 [32]	CS pain	Low	Low	Low	Low	Low	Low
Hosseinipour et al., 2024 [35]	PDPH pain	Low	Low	Low	Concern	Low	Concern
Khadem et al., 2018 [36]	Flatulence, BM	Low	Low	Low	Concern	Low	Concern
Zamani Habibabad et al., 2023 [40]	CS pain	Low	Low	Low	High	Concern	High
Postpartum							
Eradi et al., 2024 [26]	PPD	Low	Low	Low	Low	Low	Low
Chang and Chen, 2015 [33]	PPD	Low	Low	Low	Concern	Low	Concern
Nayeri et al., 2019 [37]	Sore nipples	Low	Low	Low	Concern	Low	Concern

Study Characteristics

Modes of chamomile delivery included aromatherapy or massage (eight studies) [24,28,29,31,32,38-40], tea (three studies) [23,25,33], capsules (two studies) [26,34], oral homeopathic remedy (one study) [30], and topical application (four studies) [27,35-37]. One observational study examined multiple delivery modes, including oral and topical [6]. Four observational studies did not provide specific details about the mode of chamomile delivery; two did not provide the dose of oral intake [23,26], and two did not note modes of consumption [8,9].

Three themes were observed: mode of delivery, safety, and efficacy. Each theme emerged during different phases of pregnancy. The following reviews the findings stratified by phase: during pregnancy (seven studies) [6-9,12,23,25], during labor (seven studies) [24,28-30,34,38,39], the postoperative period (six studies) [27,31,32,35,36,40], and the postpartum period (three studies) [26,33,37]. Table 5 summarizes the data from studies on the safety and efficacy of chamomile during pregnancy.

**Table 5:** Summary of the characteristics of studies evaluating efficacy and safety of chamomile during pregnancy BM: bowel movement; CH: chamomile; C/S: cesarean section; LBL: low birth length; LBW: low birth weight; M/C: miscarriage; NR: not reported; N/V: nausea/vomiting; PDPH: post-dural puncture headache; PPD: postpartum depression; PSQ: postpartum sleep quality; PTB: preterm birth; PTL: preterm labor; SGA: small for gestational age

Study	Sample size	Participant demographics	Intervention	Duration	Outcomes measured
Safety studies					
Pregnancy outcomes					
Cuzzolin et. al., 2010 [6]	392; 37 CH users	Italy, community-dwelling women, 31–40 yrs. CH PO and topical: Anxiety, digestive problems, stretch marks	Face-to-face interview, pre-structured questionnaire, self-reported data	10 - 15 min. interview Jan - Oct 2009	Higher incidence threatening M/C: 21.6%, PTL: 21.6%, SGA (general herbal use, p=0.039)
Facchinetti et al., 2012 [7]	799; 56 CH users	Italy, 3 general hospitals, age (approximately) 20 - 40 yrs. CH PO: Anxiety, constipation, stretch marks, sleep, capillary frailty, fluid retention	Face-to-face interview, pre-structured questionnaire, self-reported data	Within 3 days of childbirth, Mar 2010 - May 2011	LBW: Higher risk but not statistically significant (p=0.052); PTB, SGA: No association
Moussally and Bérard, 2012 [8]	3,183; 15 CH users	Canada (Quebec), community-dwelling women, mean age 28 yrs. CH: Delivery mode, purpose NR	Case control analysis, Quebec Pregnancy Registry, self-administered questionnaire	Oct 2006	LBW: No association
Nordeng et al., 2011 [9]	600; 29 CH users	Norway, university hospital, mean age 29.1 yrs. CH: Sedative, relaxant; delivery mode NR	Interview, structured questionnaire	5 days post-delivery, Nov 2003-Mar 2004	CH-Rx interaction: CH and psychotropic drugs (n=4, CH may increase CNS effects); no association C/S (p=0.573) or LBL (p=0.085)
Trabace et al., 2015 [12]	630; 225 CH users	Italy, public hospital, 31 - 40 yrs. CH PO: Anxiety, constipation, stretch marks, sleep, N/V	Face-to-face interview, pre-structured questionnaire	15 min. interview, 3 days post-delivery, Nov 2010-Sep 2013	PTB: Daily while pregnant, increased risk (p=0.0012); LBW: Increased risk (p=0.0183); LBL: Increased risk (p=0.0428)
Efficacy studies					
Pregnancy					
Khresheh, 2011 [23]	235; 20 CAM users	Jordan, community-dwelling women, 18 - 54 yrs	CH tea: Qualitative, open-ended question survey	2-month period	N/V: 8.5% (20/235) used CAMS; CH most useful for N/V, did not report on percentage found it useful
Forster et al., 2006 [25]	588; 65 CH users	Australia, 36–38 weeks gestation, mean 32 yrs	CH tea: Self-administered survey	No pattern observed re usage	Anxiety, sleep: 65% used; N/V, digestion: 25% used; Overall: 85% found helpful
Labor					
Pollard, 2007 [24]	196; about 90 CH users	UK, low-risk women, hospital setting, age information NR	CH topical or other oils: massage (in water bath) or aroma, oil 1% mix; audit	Duration varied by patient and practitioner	Pain: CH and lavender oil combo, reduced orthodox pain management
Heidari-fard et al., 2017 [28]	130	Iran, nulliparous pregnancy, 18-35 yrs	CH inhalation: 2 drops (1.5 g/100 mL), 7-10 cm from nose	Every 30 min, 3-4 and 8-10 cm dilation	Anxiety: Lower levels at 3-4 cm (p=0.0005), and 8-10 cm (p=0.0006)
Heidari-fard et al., 2018 [29]	130	Iran, nulliparous pregnancy, 18-35 yrs	CH inhalation: 2 drops (1.5 g/100 mL), 7-10 cm from nose	Every 30 min, 3-4 and 8-10 cm dilation	Pain: No change duration or no. contractions; lower intensity 5-7 cm (p=0.004)
Zafar et al., 2016 [30]	131	Pakistan, early labor (3-6 cm, spontaneous or induced), mean 27.3 yrs	CH PO: 3 drops homeopathic remedy + saline injection	Latent until early labor	Pain: CH PO no significant difference compared placebo or pentazocine labor pain intensity
Gholami et al., 2016 [34]	80	Iran, primipara, gestation >40 weeks, 18-36 yrs	CH PO: 6 500 mg capsules per day, 2 every 8 hrs.	Latent labor, up to 7 days	Induction: Earlier mean time to labor (p=0.003) and labor pain (p=0.00)
Eskandari et al., 2022 [38]	154	Iran, primiparous, in labor, 5 cm dilatation, mean 22 ± 3.7 yrs	CH topical: odorless oil, Swedish massage, 6, 10-minute massages	Every 20 min, 5-8, 8-10 cm dilation	Pain: Less in active and 2^nd^ stage of labor (p<0.001); shorter labor duration (p<0.05)
Eskandari et al., 2023 [39]	154	Iran, primiparous, in labor, 5 cm dilatation, mean 22 ± 3.7 yrs	CH topical: odorless oil, Swedish massage, 6, 10-minute massages	Every 20 min, 5-8, 8-10 cm dilation	Anxiety/pain: CH massage less (p<0.001)
Postoperative period					
Aradmehr et al., 2017 [27]	98	Iran, primiparous vaginal delivery with episiotomy, 18-35 yrs	CH topical: 1.3%, 0.5 gm. cream, applied to wound	2x per day, every day, for 10 days	Episiotomy: Less intense pain in CH group on days 7, 10, and 14 (p=0.03, 0.02, 0.03 respectively)
Najafi et al., 2017 [31]	80	Iran, elective cesarean, mean 30.6 ± 4.6 yrs	CH inhalation: 5% essence, 2 drops, inhale	6 hrs. for 15 min period	Cesarean: CH reduced pain scores and lowered the need for analgesics (p<0.001)
Zardosht et al., 2021 [32]	128	Iran, cesarean w/spinal anesthesia, primiparous, mean 26.1 ± 4.3 yrs	CH inhalation: 5% essence, 1 drop in cup, inhale 5 cm from nose	4, 8, 12 hrs. for 15-20 min	Cesarean: CH effectively controlling intensity of pain (p<0.01)
Hosseinipour et al., 2024 [35]	148	Iran, elective cesarean, 19-40 yrs	CH topical: 3cc cream forehead, 20 min pre- anesthesia; 2 and 4 hrs. post	After spinal anesthesia 6, 12, 24, 48 hrs.	PDPH: CH oil may reduce PDPH incidence at 6 hrs. (p=0.021) and 12 hrs. (p=0.028)
Khadem et al., 2018 [36]	142	Iran, cesarean delivery, 18-35 yrs	CH topical: 20 drops oil on abdomen after conscious and stable	Hourly 4 hrs., then every 8 hrs. till defecation	BM: CH oil less time to 1st bowel sounds (p<0.001) and BM (p<0.001)
Zamani Habibabad et al., 2023 [40]	136	Iran, elective cesarean with spinal anesthesia, 17-44 yrs	CH inhalation: CH vs. CH aroma + O_2_, 1 drop oil + 3 cc distilled water, nebulizer	At 6, 6.5, 7 hrs., 5 min	Cesarean: CH aroma reduced pain,12th and 18th hr. post-op (p=0.001)
Postpartum					
Eradi et al., 2024 [26]	128	Iran, mild/moderate PPD, 18-45 yrs	CH PO: 500 mg capsule, twice a day	8 weeks	PPD: CH reduced depression scores (p=0.002)
Chang et al., 2015 [33]	73	Taiwan, 6^th^ week postpartum, 24-43 yrs	CH tea: 1 cup (2 g dried flowers, 300 mL water, 10-15 min steep)	Immediately, 2-4 wks. follow-ups	Sleep Quality: CH improved PSQ (p=0.015); improved PPD (p=0.02), immediate term
Nayeri et al., 2019 [37]	100	Iran, exclusive breast- feeding, mean 27.4 ± 4.7 yrs.	CH topical: 1.5%, 1g, 3x/day, on areola and nipple, left on until the next breastfeed	Every 8 hours, 7 days	Sore nipples: nipple ache decreased (p<0.001)

Chamomile During Pregnancy

A survey of women in Jordan (n=235, p-value not reported) revealed that 8.5% of women used CAM to treat nausea and vomiting during pregnancy; participants reported chamomile tea as the most effective alternative treatment for managing these GI symptoms [23]. Additionally, an Australian survey (n=588, p-value not reported) concluded that of the 65 women who used chamomile during their pregnancy, 83% rated it helpful for nausea and disturbed sleep [25].

Two studies reported safety concerns regarding chamomile usage during pregnancy. Cuzzolin et al. (n=392) discovered a higher frequency of miscarriages (21.6%, p-value not reported) and an increased risk of preterm labor (21.6%, p-value not reported) and small for gestational age (p=0.039 for general herbal use) among the 37 women in this cohort who used chamomile [6]. Trabace et al. (n=630) found an increased risk for preterm birth (p=0.002), low birth weight (p=0.002), and a statistically insignificant increased risk for low birth length among 225 women who used chamomile [12].

In contrast to the above studies, Nordeng et al.'s study found no association between chamomile use and increased risk of cesarean section or low birth length among a subset of 29 women (n=600) using this herbal treatment [9]. Mousally et al. (n=3,183) found no association between chamomile use during pregnancy and low birth weight among 15 women [8]. Facchinetti et al. (n=799) found no association between chamomile use and preterm birth or gestational age; however, a statistically insignificant (p=0.05) increase in low birth weight among 56 women who reported chamomile use during pregnancy emerged [7].

Chamomile During Labor

One study (n=80) demonstrated that chamomile capsules may decrease the time to labor in post-term pregnancy (p=0.00), but the authors did not report on the safety or side effects of the treatment [34]. Another study (n=131) found that the oral intake of a liquid chamomile homeopathic remedy did not decrease labor pain compared to a placebo [30].

Two studies on the same group of women (n=154) investigating the use of chamomile oil in concert with Swedish massage during labor found that it significantly reduced both labor pain (p<0.001) and anxiety scores (p<0.001) [38,39]. A hospital audit (n=196, p-value not reported) evaluated the utilization of chamomile and lavender essential oils during therapeutic massage, baths, and aromatherapy for labor pain. This herbal therapy was associated with a reduced use of orthodox pain management during labor; however, statistical significance was not reported [24]. Heidari-Fard et al. (n=130) concluded that chamomile aroma inhalation reduced anxiety during labor at 3-4 cm dilation (p=0.0005) and 8-10 cm dilation (p=0.0006) [28]. In another study that was likely conducted on the same group of women, Heidari-Fard et al. (n=130) found that chamomile aroma inhalation also lessened contraction pain intensity at 5-7 cm dilation (p=0.004); however, no effect on contraction number or duration emerged [29].

Chamomile During the Postoperative Period

Three studies (n=136, n=128, n=80) found that chamomile aromatherapy reduced pain after cesarean section and lowered the need for conventional pain relief (p=0.001, p<0.01, p<0.001, respectively) [31,32,40]. Aradmehr et al. (n=98) observed that topical chamomile cream applied to the wound curtailed the pain of women 7-14 days after undergoing an episiotomy (p=0.03 at seven days, p=0.02 at 10 days, p=0.03 at 14 days) [27]. Hosseinipour et al. (n=148) found that the topical application of chamomile cream to the forehead may reduce postdural puncture headache in women who had received the epidural (p=0.021 at six hours and 0.028 at 12 hours) [35]. Another study (n=142) found that the application of chamomile oil to the abdomen following cesarean section appeared to speed the amount of time to first bowel sounds and bowel movements (p<0.001) [36]. 

Chamomile During the Postpartum Period

Nayeri et al. (n=100) found that chamomile topical cream applied three times daily helps reduce nipple soreness during breastfeeding (p<0.001) [37]. Two studies investigated the effects of chamomile on postpartum depression. Chang et al. (n=73) observed that chamomile tea improved the symptoms of postpartum depression in the short term (p=0.02) and that it improved postpartum sleep quality (p=0.015) [33]. Eradi et al. (n=128) found that chamomile capsules were also effective in reducing the symptoms of postpartum depression (p=0.002) [26].

Discussion

A systematic review revealed limited studies on the safety and efficacy of chamomile during peripartum and postpartum periods of pregnancy.

Potential Benefits and Risks

Benefits gleaned from the studies included in this review were as follows: pain relief during labor and following cesarean section, anxiety relief, enhanced wound healing, decreased incidence of post-dural puncture headache, increased postoperative bowel motility, improved sleep quality, nipple pain relief during breastfeeding, induction of labor in post-term pregnancy, and decreased nausea/vomiting [23-40].

A case report and conference abstract offered potential additional benefits. Silva et al. reported that consuming a chamomile infusion may increase breast milk supply (n=1), though no other reports on the galactagogue effects of chamomile were found in the literature [41]. Chamomile capsules (1,000 mg) were effective for reducing postpartum anxiety (p=0.003) in a study of 35 women, per findings shared at the International Congress on Complementary and Alternative Medicine [42].

Adverse effects reported during the randomized controlled trials assessed in this review included skin irritation and headaches associated with aromatherapy via inhalation, birthing pools, and massage [24] and nausea, headaches, and dizziness associated with oral homeopathic drops [30].

In a case report, Sridharan et al. reported that chamomile tea consumption during pregnancy led to premature constriction of the fetal ductus arteriosus in pregnant women (n=2) [43]. The administration of a chamomile-containing enema caused an anaphylactic reaction in a laboring woman (n=1), resulting in asphyxia and death of the infant [44]. Kamosillan, a chamomile-containing ointment, caused allergic contact dermatitis of the nipple in two women who used it during breastfeeding [45]. No articles were identified on the potential risk to breastfeeding infants due to maternal chamomile consumption. McGeorge et al., however, recommended that mothers wipe chamomile-containing ointments off their nipples before breastfeeding to avoid sensitizing their infants to chamomile and increasing the risk of allergic reactions related to chamomile-containing diaper creams [45].

Finally, the US FDA Adverse Events Reporting System Public Dashboard has a total of 11 adverse events reported on chamomile since 2005 including food and drug interactions, swelling, chills, fever, rash, itching, increased international normalized ratio (INR), increased blood pressure, nightmares, hallucinations, and severe reactions including hemorrhage, swollen tongue, and dyspnea [46].

Quality of Evidence

The available evidence on the safety and efficacy of chamomile usage during pregnancy is limited (experiences of 2,117 women, including case reports, conference abstract, and FDA adverse events) and weak [6-9,12,23-40,41-46]. The seven efficacy studies that were deemed high quality had very small sample sizes; thus, the level of evidence is inadequate to generalize to the general population [26-32]. Of note, the efficacy of Chamomile tea, one of the most popular herbal teas globally, was weak [13,23,25,33]. Mental health and pain outcomes appear to be most efficacious; however, the evidence draws from the experiences of only 695 women involved in high-quality studies [26-32].

**Figure 2:**
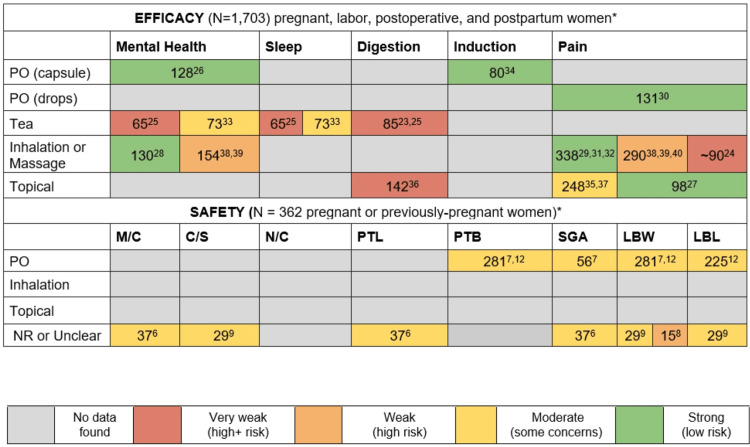
Quality of evidence stratified by study sample size, chamomile delivery mode, and symptomatology *Multiple studies with the same cohort are only counted once; only chamomile users counted in studies reporting on other herbs C/S: cesarean section; LBL: low birth length; LBW: low birth weight; M/C: miscarriage; N/C: neonatal complications; NR: not reported; PTB: preterm birth; PTL: preterm labor; SGA: small for gestational age

Except for one study with a very high risk of bias, the quality of evidence for the safety studies was moderate, reflecting some concerns regarding the risk of bias for both PO and unreported modes of delivery [6-9,12,19]. Figure 2 depicts the quality of the evidence stratified by mode of delivery and symptomatology.

Modes of delivery that appear to be efficacious include chamomile capsules, tea, topical oil (with and without massage), and inhalation [23-29,31-40]. The largest pool of data supports the efficacy of topical applications (with or without massage), albeit the combined sample size remains small (n=642, p<0.05) [27,35-39]. Topical applications with massage may also be considered a form of aromatherapy [15]. Khadem et al. reported that topical chamomile oil helped to speed up time to postoperative bowel activity after cesarean section (n=142, p<0.001) [36]. Swedish massage using chamomile oils helped significantly reduce anxiety and pain among two cohorts of pregnant women (n=130, p=0.00; n=154, p<0.001; respectively) [38,39]. Topical delivery mode (with and without massage) also offered effective pain relief [27,35,37-39].

Chamomile capsules and tea were found to be efficacious for improving mental health and provoking induction (n=128, p=0.002; n=73, p=0.02; n=80, p=0.00; respectively) [26,33,35]. Chang et al. also found that chamomile tea helped manage sleep disturbances among pregnant women (n=73, p=0.02) [33]. The findings of one efficacy study investigating chamomile drops PO for mitigating the intensity of labor pain (latent to early labor) were not significant [30].

**Figure 3:**
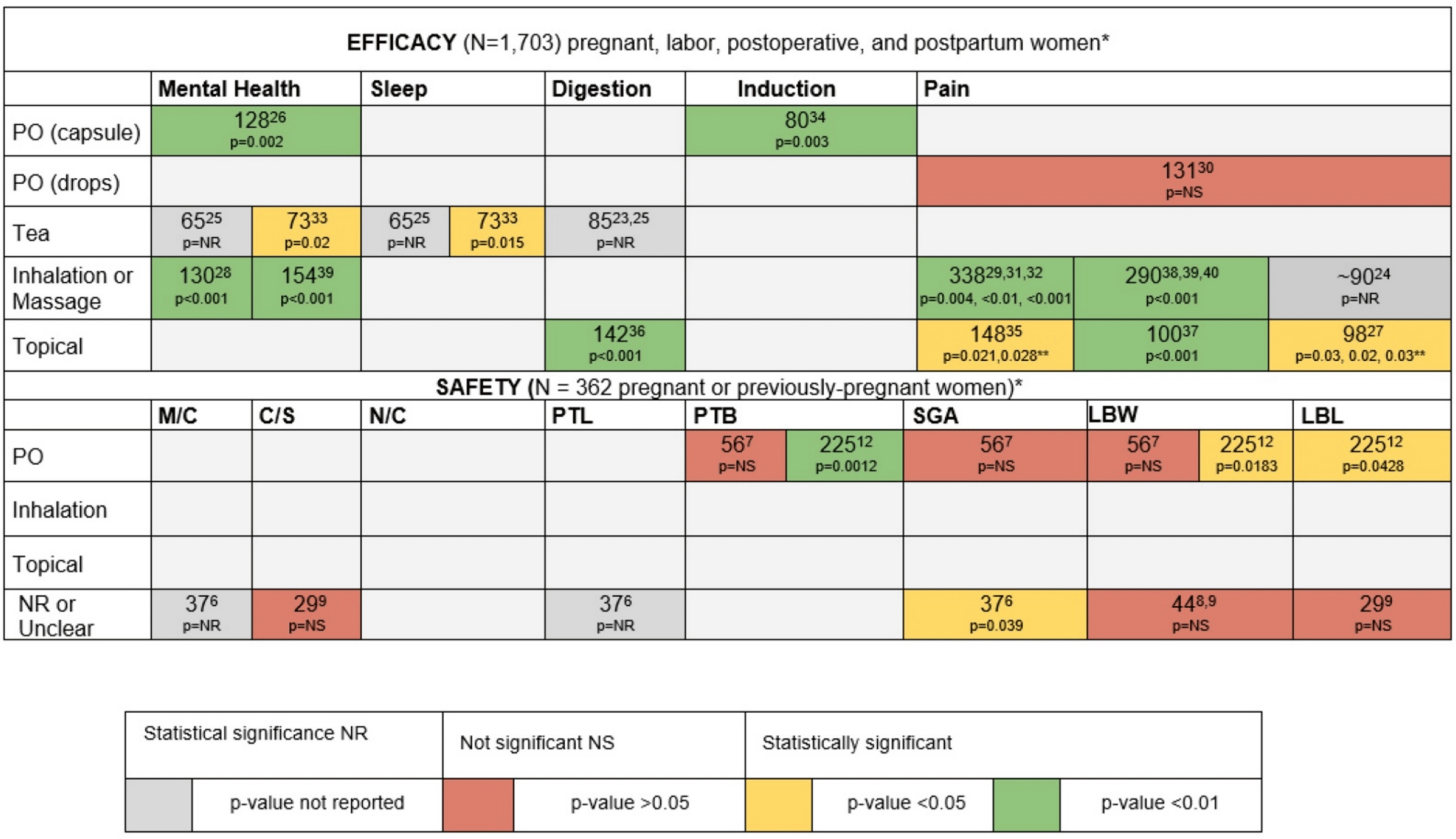
Statistical significance of evidence on efficacy (n=1,703) and safety (n=362) stratified by study sample size, chamomile delivery mode, and symptomatology *Multiple studies with the same cohort are only counted once; only chamomile users counted in studies reporting on other herbs. **Multiple p-values reflect measurements taken at different time intervals C/S: cesarean section; LBL: low birth length; LBW: low birth weight; M/C: miscarriage; NR: not reported; NS: not significant; PTB: preterm birth; PTL: preterm labor; SGA: small for gestational age

Only one safety study reported statistically significant results [12]. Based on in-person interviews (n=220) and a non-validated questionnaire, the authors found that consumption of chamomile PO significantly increased the risk of preterm birth (p=0.002) and low birth weight babies (p=0.002). Figure 3 summarizes the statistical significance of the evidence stratified by mode of delivery and symptomatology.

These findings mirror those of Vora et al. [15]. In a review of clinical aromatherapy, the authors concluded the need for more rigorous research protocols and comprehensive reporting [15]. In a review of herbal teas consumed during pregnancy, Terzioglu Bebitoglu concludes that herbal teas and infusions are safe in moderation but warns that chamomile and other herbal products consumed during pregnancy may contain toxic contaminants and yield adverse maternal and perinatal outcomes [13].

Translation of Findings into Clinical Practice

Based on the available evidence, it is premature to consider clinical practice recommendations for using chamomile products during pregnancy. If a patient wants to use chamomile therapeutically during pregnancy, labor, or postpartum, a risk-benefit counseling session based on the evidence in the existing literature is recommended.

During the patient counseling session, the information to review is that herbal supplements are not regulated as strictly as pharmaceuticals [47]. Dietary supplements are only subject to post-market regulation [47]. In the US, manufacturers can produce and sell a dietary supplement without notifying the US Food and Drug Administration as long as there are no “new dietary ingredients” [47]. Additionally, the dosage or serving size listed on the packages may not be supported by clinical trials; therefore, doses vary from manufacturer to manufacturer [47]. Thus, it can be challenging to know the exact exposure in a specific supplement, the potential contaminants present, or the accuracy of the product labeling. Another consideration is the potential for herb-drug interactions between chamomile and psychotropics, sedatives, birth control, and blood thinners [9,48].

An important skill for providers is distinguishing between the clinical significance of treatment and the statistical significance of research findings. Clinically significant results demonstrate cost-effectiveness and superior outcomes when compared to other agents that are already available to patients [49]. Chamomile products can also improve the quality of patients’ lives, yielding the costs, potential harms, and inconveniences of the treatment worth the benefit [49]. Thus, despite weak evidence supporting the efficacy and safety of chamomile usage during pregnancy, providers might share the potential benefits and risks with their patients. Table 6 provides a breakdown of key points for a patient counseling session.

**Table 6:** Evidence-based guidance for a patient counseling session regarding chamomile usage during pregnancy FDA: Food and Drug Administration

Key points for a patient counseling session regarding chamomile use	
Overall, it is unknown if chamomile is helpful or safe. The safety and needs of both the mother and fetus need to be considered before opting to use chamomile during pregnancy	
The level of evidence for the safety and efficacy of chamomile during and following pregnancy is weak	
Chamomile tinctures may contain up to 12% alcohol, which should be used with caution during pregnancy [1,50]	
Risks during pregnancy: miscarriage, preterm labor/birth, small for gestational age, low birth weight, low birth length [6,7,12]	
Drug interactions: psychotropics, sedatives, birth control, blood thinners [9,48]	
During pregnancy, chamomile may alleviate symptoms such as nausea and vomiting [23,25]	
During labor and following cesarean section, chamomile aromatherapy and topical application may reduce pain and anxiety [24,28,29,31,32,38-40]	
Reported adverse effects: skin irritation/allergic contact dermatitis, headache, nausea, dizziness, anaphylaxis, fever, nightmares, hallucinations [24,30,44-46]	
Postpartum, chamomile may improve depression, anxiety, and sleep quality [25,26,33,42]	
Chamomile is regulated by the FDA as a dietary supplement and is subject to post-market regulation [47]	

Strengths and Limitations

Strengths of this review include the tools employed and the exclusion of research published in the grey literature. Other strengths were the involvement of an interprofessional duo of researchers and the analysis of a diverse pool of women from around the world. Limitations include a small sample size plus the inclusion of multiple studies by the same research teams, e.g., Eskandari et al., 2022 and 2023, and Heidari-Fard et al., 2017 and 2018 [28,29,38,39]. These findings likely reflect insights from the same cohort of women. Another limitation was that the range of chamomile types, doses, and delivery modes restricted the robustness of the comparison of studies. A meta-analysis was not conducted for this reason. Additionally, the restriction of studies only published in English may have excluded research from countries where chamomile products have high usage rates.

## Conclusions

Our findings point to a lack of strong evidence supporting the use of chamomile during pregnancy. Patient education on the potential toxicities of herbal products and the risk for adverse maternal and perinatal outcomes associated with high levels of consumption is imperative. The findings also highlight the importance of improved dissemination of the potential adverse effects of herbal remedies to healthcare practitioners, as well as the need for providers to query patients about their CAM usage.

Further research on the safety and efficacy of chamomile during the peripartum and postpartum periods is needed, and more rigorous research is critical to confirm its safety and efficacy during pregnancy. Gaps in the literature include the prevalence of chamomile use in the US, contaminants present in chamomile supplements and teas, adverse reactions to chamomile, physician knowledge and attitudes surrounding herbal product use, and the prevalence of chamomile usage with concomitant medications. The need for randomized, double-blind placebo control studies with larger study populations and consistent study protocols emerged. To find deeper and definitive insights into the safety and efficacy during pregnancy, clinical trials should employ consistent study methodologies and modes of chamomile delivery, reporting on the type and origin of chamomile, the inclusion of more diverse and larger sample sizes, and uniform reporting of adverse reactions.
